# Diagnostic and prognostic value of speckle tracking echocardiography for right ventricular dysfunction in sepsis: a retrospective observational study

**DOI:** 10.1186/s12872-025-05175-9

**Published:** 2025-10-06

**Authors:** Yao Yao, Qingnan Yang, Ming Zhong, Hongyu He, Jieqiong Song

**Affiliations:** 1 Department of Critical Care Medicine, Zhongshan Hospital, Fudan University, Shanghai, 200030 China; 2 Department of Critical Care Medicine, Shanghai Geriatrics Medical Center, Shanghai, 201104 China

**Keywords:** Sepsis, Right ventricular dysfunction, Speckle tracking echocardiography, Longitudinal strain, Tissue motion annular displacement

## Abstract

**Background:**

Right ventricular dysfunction (RVD) is increasingly recognized as a prognostic factor in sepsis. The diagnostic accuracy and prognosis value of speckle tracking echocardiography (STE)-derived parameters such as right ventricular longitudinal strain (RVLS) and tissue motion annular displacement (RVTMAD) remain inadequately defined in critically ill patient.

**Methods:**

This retrospective observational study included patients admitted to the intensive care unit (ICU) with sepsis or septic shock from March 2019 to November 2021. Transthoracic echocardiography was performed within 24 h of ICU admission. RVLS and RVTMAD were measured offline using STE. The diagnostic performance of these parameters for RVD was evaluated using ROC curve analysis, and their association with mortality was assessed using Cox regression models.

**Results:**

Among 159 enrolled patients, 57 (35.8%) were diagnosed with RVD. Patients with RVD exhibited significantly higher (less negative) RVLS (*P* < 0.05). RVTMADmid and RVTMAD% demonstrated the highest diagnostic accuracy for RVD, with AUROC values of 0.913 (95% CI: 0.866–0.960) and 0.917 (95% CI: 0.874–0.960), respectively. Patients with RVTMADmid < 10.95 mm or RVTMAD% < 16.70%, had higher in-hospital mortality. Right ventricular free-wall longitudinal strain (RVFWLS) ≥ − 18.85% was associated with higher in-hospital and 28-day mortality. In the Cox proportional hazards regression analysis, the SOFA score emerged as the sole independent predictor of in-hospital mortality.

**Conclusion:**

RVTMAD is a simple and reproducible STE-derived parameter with excellent diagnostic performance for identifying RVD in sepsis. It may serve as a valuable complement to conventional echocardiographic measures, particularly when image quality is suboptimal.

**Supplementary Information:**

The online version contains supplementary material available at 10.1186/s12872-025-05175-9.

## Introduction

Sepsis is a life-threatening condition characterized by a dysregulated host response to infection, frequently leading to multi-organ dysfunction and high mortality [1]. While sepsis-induced cardiomyopathy (SICM) has historically focused on left ventricular (LV) dysfunction [2, 3], growing evidence suggests that right ventricular (RV) dysfunction (RVD) is both common and prognostically significant in this population [4–6].

Accurate assessment of RV function in septic patients remains clinically challenging. Conventional echocardiographic parameters such as tricuspid annular plane systolic excursion (TAPSE) and fractional area change (FAC) are commonly used to evaluate RV systolic function but suffer from technical limitations, including angle dependency and load sensitivity [7, 8]. However, in patients with significant tricuspid regurgitation (TR), RV assessment is complicated by the interplay between volume overload caused by TR and resultant RV dilatation and dysfunction. This bidirectional relationship can obscure the accurate interpretation of RV functional indices [9]. Moreover, the complex RV geometry and poor acoustic windows in critically ill patients often result in suboptimal image quality [10, 11]. Therefore, a comprehensive evaluation that considers both the severity of TR and RV structural and functional parameters is essential.

Two-dimensional speckle tracking echocardiography (STE) is an advanced imaging technology that allows for the quantitative assessment of myocardial deformation, offering a more detailed evaluation of ventricular systolic function [12]. STE has been extensively utilized in cardiology for diagnosing and managing various cardiomyopathies [13, 14]. Compared to traditional echocardiographic parameters, RV longitudinal strain (RVLS) derived from STE is angle-independent, less affected by loading conditions, and provides a more accurate representation of RV function (15,16).

Additionally, tissue motion annular displacement (TMAD) is a novel semi-automated STE-derived parameter that quantifies annular motion and has shown promise in assessing ventricular function. Unlike conventional RV parameters, RVTMAD is less reliant on image quality and is also angle-independent [17]. While LVTMAD has been successfully applied in differentiating SICM [18], the clinical utility of RVTMAD in critically ill patients remains largely unexplored. To date, limited data exist regarding the diagnostic and prognostic value of RVLS and RVTMAD in septic patients.

Therefore, this study aimed to evaluate the diagnostic accuracy of STE-derived RVLS and RVTMAD for identifying RVD in sepsis and to explore their potential prognostic value in septic patients.

## Methods

### Study design and patients

This retrospective observational study was conducted at Zhongshan Hospital, Fudan University, and included patients admitted to the intensive care unit (ICU) between March 2019 and November 2021. The Ethics Committee of Zhongshan Hospital, Fudan University, approved the project (approval #B2021-501R). The requirement for individual informed consent was waived because no interventions were performed, there was no contact with the patients, and all data were retrospectively and anonymously collected. The present study was conducted and reported according to the STROBE statement [19].

The inclusion criteria were (1) age ≥ 18 years, (2) diagnosed with sepsis or septic shock [20], and (3) underwent echocardiography examination within 24 h of ICU admission. The patients who met any of the following exclusion criteria were excluded: (1) incomplete echocardiographic data or poor image quality, (2) history of heart failure due to coronary heart disease, cardiomyopathy, or pulmonary heart disease, (3) all degrees of valvular heart disease, (4) atrial fibrillation, or (5) use of a cardiac implanted device.

### Data collection

The basic characteristics and clinical data were collected retrospectively from the electronic medical record system based on the admission data. Basic characteristics encompassed age, sex, body mass index (BMI), surgery site, sepsis source, Charlson comorbidity index. Clinical data collected include Acute Physiology and Chronic Health Evaluation II (APACHE II) score, Sequential Organ Failure Assessment (SOFA) score, heart rate (HR), mean arterial pressure (MAP), central venous pressure (CVP), receipt of mechanical ventilation (MV) at time of echocardiography, and norepinephrine base (NE) dosage. Laboratory information included cardiac troponin T (cTnT), N-terminal pro-brain natriuretic peptide (NT-proBNP), alanine transaminase (ALT), aspartate transaminase (AST), creatinine (Cr), and PaO2/FiO2 ratio (P/F). The occurrence of acute respiratory distress syndrome (ARDS), acute kidney injury (AKI), duration of MV, in-hospital stay, ICU stay, 28-day survival, and in-hospital survival were collected from the medical charts. ARDS was defined according to the criteria proposed in the 2024 publication “A New Global Definition of Acute Respiratory Distress Syndrome” [21]. AKI was defined according to the Kidney Disease Improving Global Outcomes (KDIGO) clinical practice guidelines [22].

### Routine transthoracic echocardiography

Data about transthoracic echocardiography examinations were collected from the patient charts. During the study period, all examinations were performed using a CX50 CompactXtreme Ultrasound System (Philips, MA, USA) equipped with a 1–5 MHz-phased array transducer. The following echocardiographic parameters were extracted from the patient charts or previously stored ultrasound images: left ventricular ejection fraction (LVEF), cardiac output (CO), mitral annular plane systolic excursion (MAPSE), right atrial (RA) and RV dimension, FAC, TAPSE, and pulmonary arterial systolic pressure (PASP), TAPSE/PASP, and RV-to-LV end-diastolic area ratio (RV/LVEDV). LVEF was quantified as a percentage using the Simpson biplane method. RV dimensions, including basal and longitudinal diameters, were measured according to current guidelines [7]. RV end-systolic and end-diastolic areas were traced in the apical four-chamber view to calculate FAC using the following formula: FAC = ([diastolic area − systolic area]/diastolic area) × 100% [10]. TAPSE represented the maximal displacement of the tricuspid annulus on M-mode in the apical 4-chamber view [10]. PASP was calculated using the Bernoulli equation with tricuspid regurgitant maximum velocity (TRV): (4 × TRV²) + RAP [22]. In this study, RVD was defined as FAC < 35% or TAPSE < 16 mm [10].

### Speckle tracking echocardiography

**Fig. 1:**
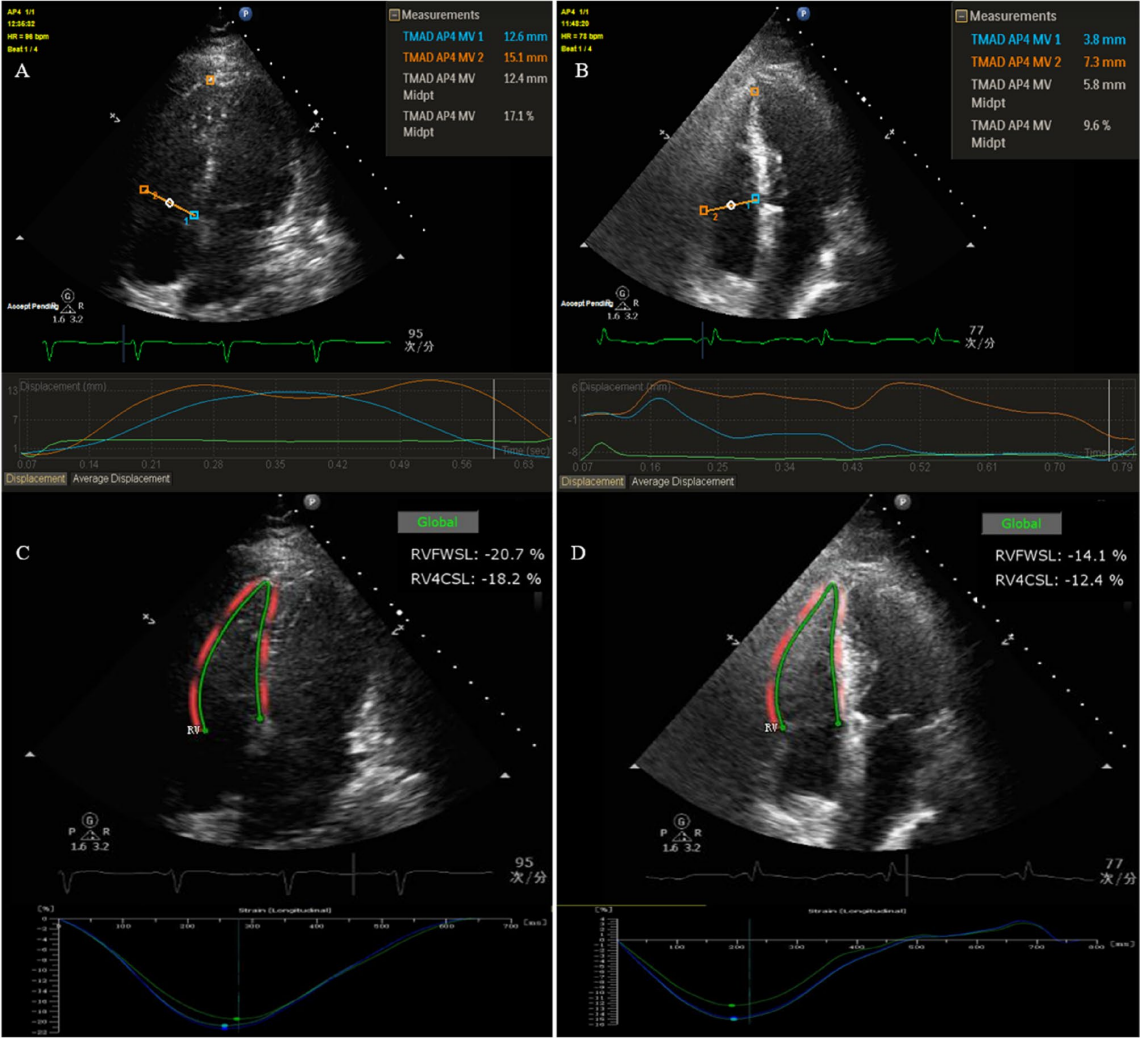
Speckle tracking echocardiography (STE) images of septic patients with or without right ventricular dysfunction (RVD). **A** Four-chamber view of the right ventricular (RV) tissue motion annular displacement (RVTMAD) measurement of one patient with normal RV function. **B** Four-chamber view of the RV tissue motion annular displacement (RVTMAD) measurement of one patient with right ventricular dysfunction (RVD). **C** four-chamber view of the RV longitudinal strain (RVLS) measurement of one patient with normal RV function. **D** Four-chamber view of the RV longitudinal strain (RVLS) measurement of one patient with RV dysfunction (RVD)

The STE offline analyses were performed by two independent investigators who were proficient in echocardiography software. During the image review, both investigators were blinded to all clinical data. RVLS and RV tricuspid annular motion amplitude displacement (RVTMAD) were analyzed using Philips QLAB software, version 17.3 (Philips Healthcare, MA, USA). For RVLS analysis, apical 4-chamber views displaying the best available single cardiac cycle with optimal endocardial image quality were chosen [23, 24]. The software automatically tracked the RV endocardial border to generate RV 4-chamber longitudinal strain (RV4CLS) and RV free-wall longitudinal strain (RVFWLS). The operator could manually adjust the endocardial borders for adequate tracking quality (Fig. 1).

RVTMAD evaluated the displacement of the tricuspid annulus toward the apex during the cardiac cycle. It was quantified by placing points of interest on the lateral and septal tricuspid annuli, with a reference point at the RV apex [17]. After manually defining these three points, RVTMAD septal (RVTMAD1), lateral (RVTMAD2), midpoint (RVTMADmid), and RVTMAD% were automatically generated. RVTMAD1 and RVTMAD2 reflected the movement of the selected annular points toward the RV apex in a straight line. RVTMADmid was calculated by the QLAB software using a point that bisected the line between the annular points. RVTMAD% represented the midpoint displacement divided by the total length of the RV [25].

Only images with a frame rate ≥ 50 frames per second were selected to ensure analysis reliability. Echocardiographic data were recorded over three consecutive cardiac cycles and then averaged.

The acquisition rates of FAC, TAPSE, RVLS, and RVTMAD were individually calculated. To assess the time required for measurement and reproducibility, a subset of 20 patients was randomly selected. The duration of offline analyses for FAC, TAPSE, RVLS, and RVTMAD was recorded. Intra-observer reproducibility was evaluated by having the same investigator remeasure the same 20 patients 4 weeks after the initial evaluation. Inter-observer reproducibility was assessed by having a second investigator independently examine the images of the same 20 patients. Both investigators were blinded to the original measurements. Intra- and inter-observer reproducibility were evaluated using the intraclass correlation coefficient (ICC), which ranges from 0 to 1. ICC scores below 0 were considered unreliable, while scores of 0.75 or higher indicated acceptable reliability.

### Study outcomes

The primary outcome of this study was the diagnostic accuracy of STE-derived parameters, specifically RVLS and RVTMAD, in detecting RVD in patients with sepsis.

The secondary outcomes included in-hospital mortality and 28-day mortality, defined respectively as death occurring during hospitalization and death within 28 days of hospital admission.

### Statistical analysis

According to the primary outcomes of the study, the expected sensitivity and specificity of STE in the diagnosis of RVD were 90% and 85%. The prevalence of RVD in patients with sepsis is approximately 33% [4, 5]. The sample size was calculated using confidence intervals for the one-sample sensitivity and specificity with α = 0.05 and β = 0.10. Considering a loss to follow-up rate of about 2%, the sample size was 50 patients in the RVD group, and 100 patients in the non-RVD group.

The continuous variables were presented as means ± standard deviation of the mean (SEM) or median [interquartile range]. Student’s t-test was used for normally distributed continuous variables, whereas the Wilcoxon signed-rank test was used for non-normally distributed variables. The categorical variables were presented as n (%) and analyzed using the chi-squared test or Fisher’s exact test. Pearson’s correlation analysis was conducted to assess the correlation between conventional echocardiography parameters and STE measurements. The optimal cutoff values for RVLS and RVTMAD in diagnosing RVD were determined using the area under the receiver operating characteristic (ROC) curves (AUROCs) based on the maximum Youden index. Kaplan-Meier curves and the log-rank test were used to analyze the prognostic value of these echocardiographic parameters. Cox proportional hazard model analysis was performed to explore the prognostic value of variables for 28-day and in-hospital mortality. Variables with *P* < 0.05 in the univariable analyses were included in the Cox regression using stepwise forward selection. To evaluate potential collinearity among covariates, a correlation matrix was constructed. Variables exceeding 0.3 were carefully reviewed to determine which to retain in order to avoid multicollinearity. Clinically relevant variables with low intercorrelation would be included in the Cox proportional hazards model. Statistical analysis was conducted using SPSS 20.0 (IBM, Armonk, NY, USA). Graphs were created using GraphPad Prism 9.0 (GraphPad Software, La Jolla, CA, USA). P-values < 0.05 were considered statistically significant.

## Results

### Patients characteristics

**Fig. 2:**
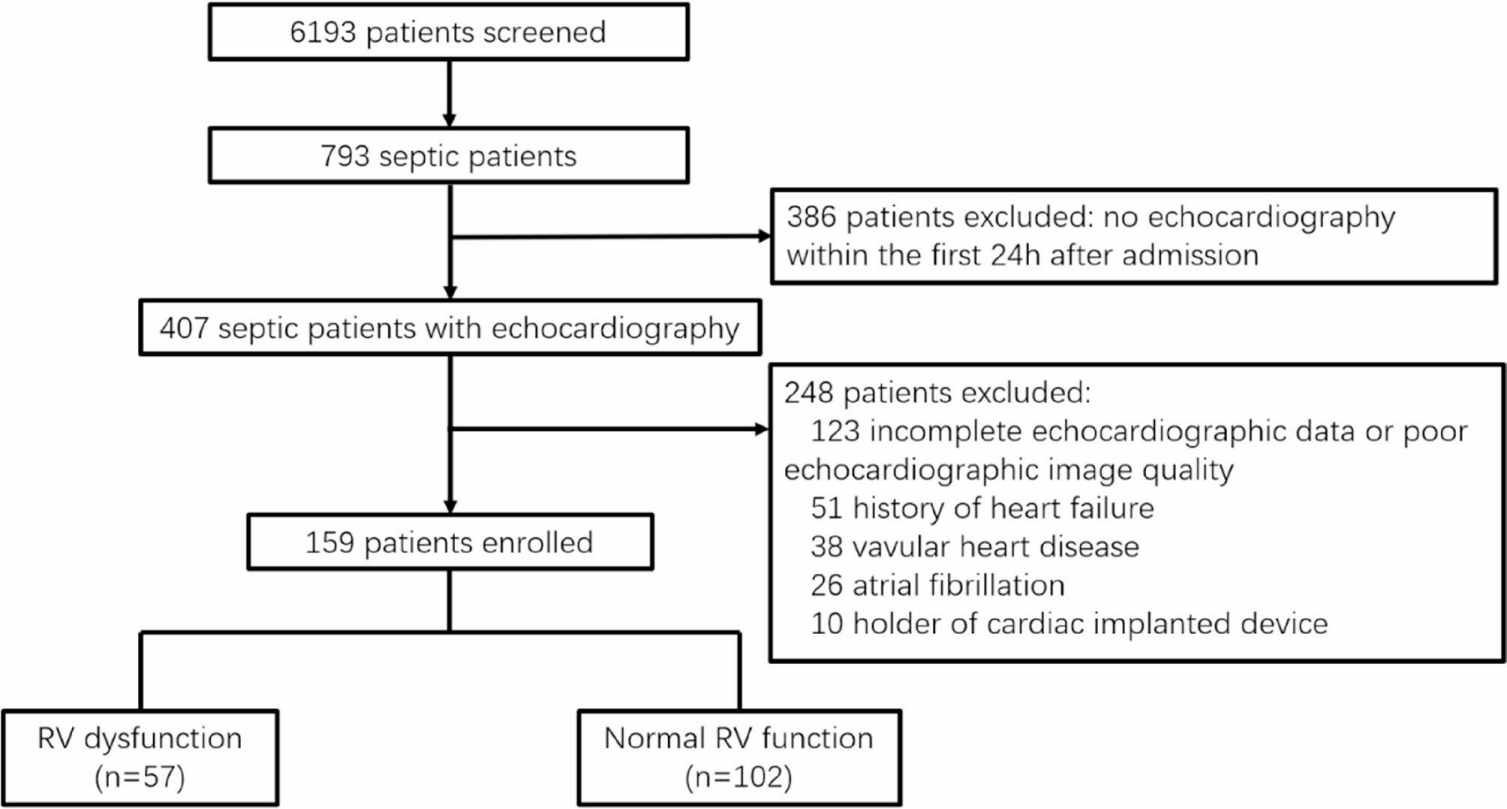
The flow diagram detailing the enrollment of patients in the study

Between March 2019 and November 2021, 6,193 patients were admitted to the ICU, among whom 793 were diagnosed with sepsis. Transthoracic echocardiograms were performed within the first 24 h of ICU admission in 407 patients. After applying the inclusion and exclusion criteria, 159 patients were included in the final analysis, comprising 57 patients with RVD and 102 without (Fig. 2).

Baseline characteristics are summarized in Table 1. The median age of the cohort was 66 years, with 62.9% being male. There were no significant differences between the RVD and non-RVD groups in terms of age, sex, BMI, SOFA score, surgery site, infection source or ARDS incidence. However, patients with RVD had significantly higher APACHE II score (17.8 ± 0.8 vs. 16.0 ± 0.5, *P* = 0.048) and a higher incidence of AKI (47.4% vs. 24.5%, *P* = 0.003). Laboratory findings revealed significantly elevated levels of cTnT, NT-proBNP, ALT, and AST in the RVD group compared to the non-RVD group (all *P* < 0.05). In-hospital mortality was also significantly higher in the RVD group (31.6% vs. 15.7%, *P* = 0.019).

**Table 1:** Characteristics of the patients

Characteristics	All participants (*n* = 159)	RVD (*n* = 57)	Non-RVD (*n* = 102)	*P*
Age (years)	66.6 ± 1.3	68.3 ± 2.2	65.6 ± 1.6	0.316
Sex (Male, %)	100, 62.9	35, 61.4	65, 63.7	0.771
BMI (kg/m^2^)	23.2 ± 0.3	22.9 ± 0.3	23.3 ± 0.4	0.439
Surgery (n, %)				0.926
Abdominal	75 (47.2)	29 (50.9)	46 (45.1)	
Thoracic	38 (23.9)	14 (24.6)	24 (23.5)	
Cranial	30 (18.9)	9 (15.8)	21 (20.6)	
Urological	13 (8.2)	4 (7.0)	9 (8.8)	
Others	3 (1.9)	1 (1.8)	2 (2.0)	
Sepsis source (n, %)				0.975
Abdominal	71 (44.7)	28 (49.1)	43 (42.2)	
Pneumonia	44 (27.7)	15 (26.3)	29 (28.4)	
Urinary tract	20 (12.6)	7 (12.3)	13 (12.7)	
Bloodstream	13 (8.2)	4 (7.0)	9 (8.8)	
Catheter	6 (3.8)	2 (3.5)	4 (3.9)	
Others	5 (3.1)	1 (1.8)	4 (3.9)	
Charlson comorbidity index	6 [4–10]	7 [4–10]	6 [3–10]	0.766
APACHE II	16.4 ± 0.5	17.8 ± 0.8	16.0 ± 0.5	0.048
SOFA	6.6 ± 0.2	7.1 ± 0.5	6.4 ± 0.2	0.124
HR (bpm)	99.4 ± 1.6	103.0 ± 2.5	97.3 ± 2.0	0.078
CVP (mmHg)	9.8 ± 0.5	11.0 ± 0.5	8.9 ± 0.5	0.050
CK-MB (ng/mL)	7.7 ± 1.3	9.5 ± 2.7	5.9 ± 0.8	0.051
cTNT (ng/mL)	0.09 ± 0.02	0.16 ± 0.05	0.06 ± 0.01	0.009
NT-proBNP (pg/mL)	3773 ± 561	6899 ± 1359	1911 ± 234	< 0.0001
ALT (U/L)	90.6 ± 15.9	137.5 ± 38.7	62.8 ± 9.9	0.022
AST (U/L)	166.4 ± 41.9	289.8 ± 108.5	93.5 ± 14.9	0.023
P/F ratio (mmHg)	252.7 ± 9.3	236.1 ± 14.0	262.5 ± 12.3	0.173
Septic shock (n, %)	118 (74.2)	45 (78.9)	73 (71.6)	0.308
NE (µg/kg/min)	0.19 ± 0.01	0.21 ± 0.02	0.18 ± 0.02	0.232
Cr (µmol/L)	80.6 ± 4.2	85.4 ± 8.2	77.9 ± 4.6	0.392
ARDS (n, %)	76 (47.8)	29 (51.8)	47 (46.1)	0.510
AKI (n, %)	52 (32.7)	27 (47.4)	25 (24.5)	0.003
Receipt of MV (n, %)	146 (91.8)	52 (91.2)	94 (92.2)	0.838
MV duration (hours)	36 [11–126]	41 [16–169]	31 [11–101]	0.078
In-hospital stay (days)	18 [12–29]	18 [13–30]	18 [12–29]	0.937
ICU stay (days)	6 [3–12]	7 [4–18]	6 [3–10]	0.196
28-day mortality (n, %)	28 (17.6)	14 (24.6)	14 (13.7)	0.085
In-hospital mortality (n, %)	34 (21.4)	18 (31.6)	16 (15.7)	0.019

*RVD* right ventricular dysfunction, *BMI* body mass index, *APACHE II* acute physiology and chronic health evaluation II, *SOFA* sequential organ failure assessment, *HR* heart rate, *CVP* central venous pressure, *CK-MB* creatine kinase isoenzyme, *cTnT* cardiac troponin T, *NT-proBNP* N-terminal pro-B type natriuretic peptide, *ALT* alanine transaminase *AST* aspartate transaminase, *Cr* creatinine, *P/F* PaO2/FiO2 ratio, *NE* norepinephrine base, *MV* mechanical ventilation, *AKI* acute kidney injury

### Echocardiographic findings

Conventional and STE-derived echocardiographic parameters are detailed in Table 2. Patients in the RVD group exhibited significantly lower values for LVEF, MAPSE, CO, FAC, TAPSE, TAPSE/PASP ratio, and RVTMAD, compared to those without RVD (all *P* < 0.05). As expected, RVLS values were significantly higher (less negative) in the RVD group (all *P* < 0.05).

**Table 2:** Echocardiography data of the patients

Variables	All participants	RVD (*n* = 57)	Non-RVD (*n* = 102)	*P*
HR (bpm)	99.4 ± 1.6	103.0 ± 2.5	97.3 ± 2.0	0.082
MAP (mmHg)	69 ± 1.1	64 ± 0.9	76 ± 1.2	0.194
LVEF (%)	56.9 ± 0.7	52.3 ± 1.4	59.2 ± 0.7	< 0.0001
MAPSE (mm)	12.7 ± 0.3	11.0 ± 0.5	13.6 ± 0.4	< 0.0001
CO (L/min)	4.81 ± 0.14	4.29 ± 0.21	5.12 ± 0.17	0.003
RA diameter (mm)	42.6 ± 1.0	43.8 ± 1.5	41.4 ± 0.8	0.118
RV basal diameter (mm)	32.9 ± 0.9	32.9 ± 1.1	32.9 ± 0.9	0.984
RV longitudinal diameter (mm)	63.0 ± 1.2	61.9 ± 1.2	65.4 ± 1.2	0.056
RV/LVEDV	0.33 ± 0.08	0.32 ± 0.09	0.35 ± 0.12	0.791
FAC (%)	40.8 ± 0.8	32.3 ± 1.2	45.5 ± 0.8	< 0.001
TAPSE (mm)	19.2 ± 0.4	15.7 ± 0.6	21.4 ± 0.4	< 0.001
PASP (mmHg)	31.7 ± 1.1	32.9 ± 1.5	30.5 ± 1.3	0.064
TAPSE/PASP	0.74 ± 0.03	0.56 ± 0.04	0.84 ± 0.04	< 0.001
RVTMAD1 (mm)	10.3 ± 0.3	7.2 ± 0.4	12.0 ± 0.4	< 0.001
RVTMAD2 (mm)	15.1 ± 0.5	10.5 ± 0.5	17.7 ± 0.5	< 0.001
RVTMADmid (mm)	12.5 ± 0.4	8.5 ± 0.4	14.7 ± 0.4	< 0.001
RVTMAD%	17.1 ± 0.5	12.0 ± 0.5	19.8 ± 0.5	< 0.001
RV4CLS (%)	−13.3 ± 0.4	−10.4 ± 0.5	−15.0 ± 0.5	< 0.001
RVFWLS (%)	−16.1 ± 0.5	−12.5 ± 0.7	−18.3 ± 0.6	< 0.001

*RV* right ventricle, *RVD* right ventricular dysfunction, *HR* heart rate, *MAP* Mean arterial pressure, *RVLS* right ventricular longitudinal strain, *RV/LVEDV* RV-to-LV end-diastolic area ratio, *FAC* fractional area change, *LVEF* left ventricle ejection fraction, *TMAD* tissue motion annular displacement, *RVTMAD* right ventricular tricuspid annular motion amplitude displacement, *RV4CLS* RV 4-chamber longitudinal strain, *RVFWLS* RV free-wall longitudinal strain, *RA* right atrium, *MAPSE* mitral annular plane systolic excursion, *TAPSE* tricuspid annular plane systolic excursion, *PASP* pulmonary arterial systolic pressure, *CO* cardiac output

**Fig. 3:**
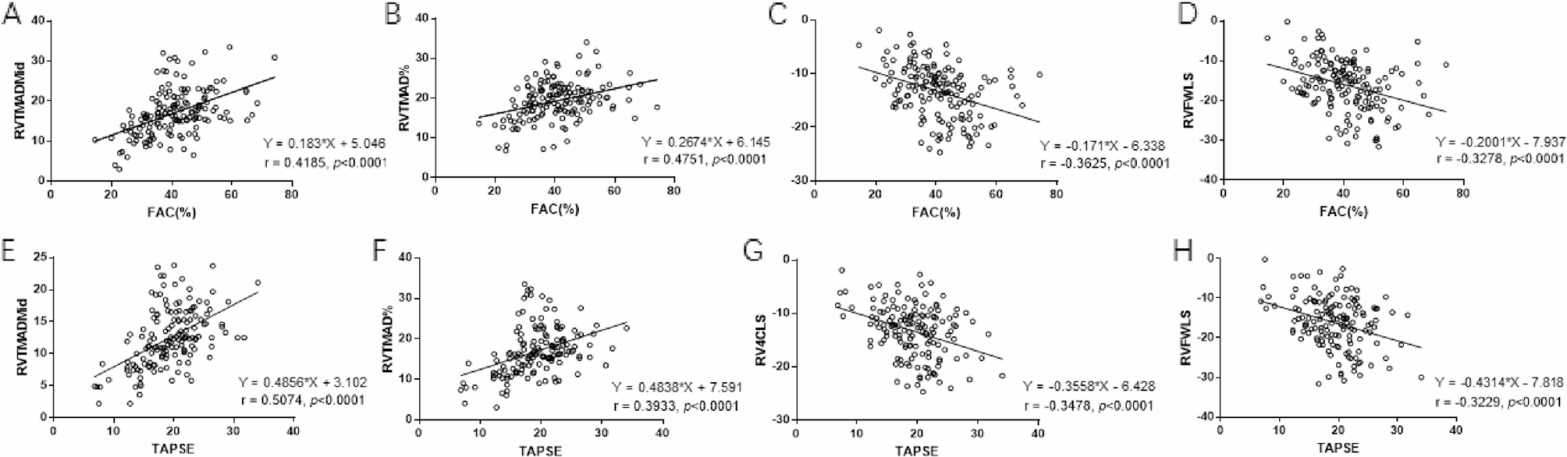
Relationship between conventional echocardiographic parameters and speckle tracking echocardiography (STE) parameters. **A** Positive correlation between fractional area change (FAC) and RV tissue motion annular displacement (RVTMAD)mid. **B** Positive correlation between fractional area change (FAC) and RV tissue motion annular displacement (RVTMAD)%. **C** Negative correlation between fractional area change (FAC) and RV 4-chamber longitudinal strain (RV4CLS). **D** Negative correlation between fractional area change (FAC) and RV free-wall longitudinal strain (RVFWLS). **E** Positive correlation between tricuspid annular plane systolic excursion (TAPSE) and RV tissue motion annular displacement (RVTMAD)mid. **F** Positive correlation between tricuspid annular plane systolic excursion (TAPSE) and RV tissue motion annular displacement (RVTMAD)%. **G** Negative correlation between tricuspid annular plane systolic excursion (TAPSE) and RV 4-chamber longitudinal strain (RV4CLS). **H** Negative correlation between tricuspid annular plane systolic excursion (TAPSE) and RV free-wall longitudinal strain (RVFWLS)

Correlation analysis demonstrated strong positive correlations between RVTMAD (mid and %) and conventional RV function indices (FAC and TAPSE), while RVLS (RV4CLS and RVFWLS) showed significant negative correlations with both FAC and TAPSE (all *P* < 0.001) (Fig. 3). These findings suggest that STE parameters reflect established markers of RV dysfunction.

Intra- and inter-observer variability were assessed using the ICCs. As shown in TableS1, both RVLS and RVTMAD demonstrated excellent reproducibility, with ICC values exceeding 0.85 for all comparisons. Notably, RVTMAD showed the highest ICCs, indicating superior measurement consistency.

### Diagnostic accuracy of STE parameters for RVD

**Fig. 4:**
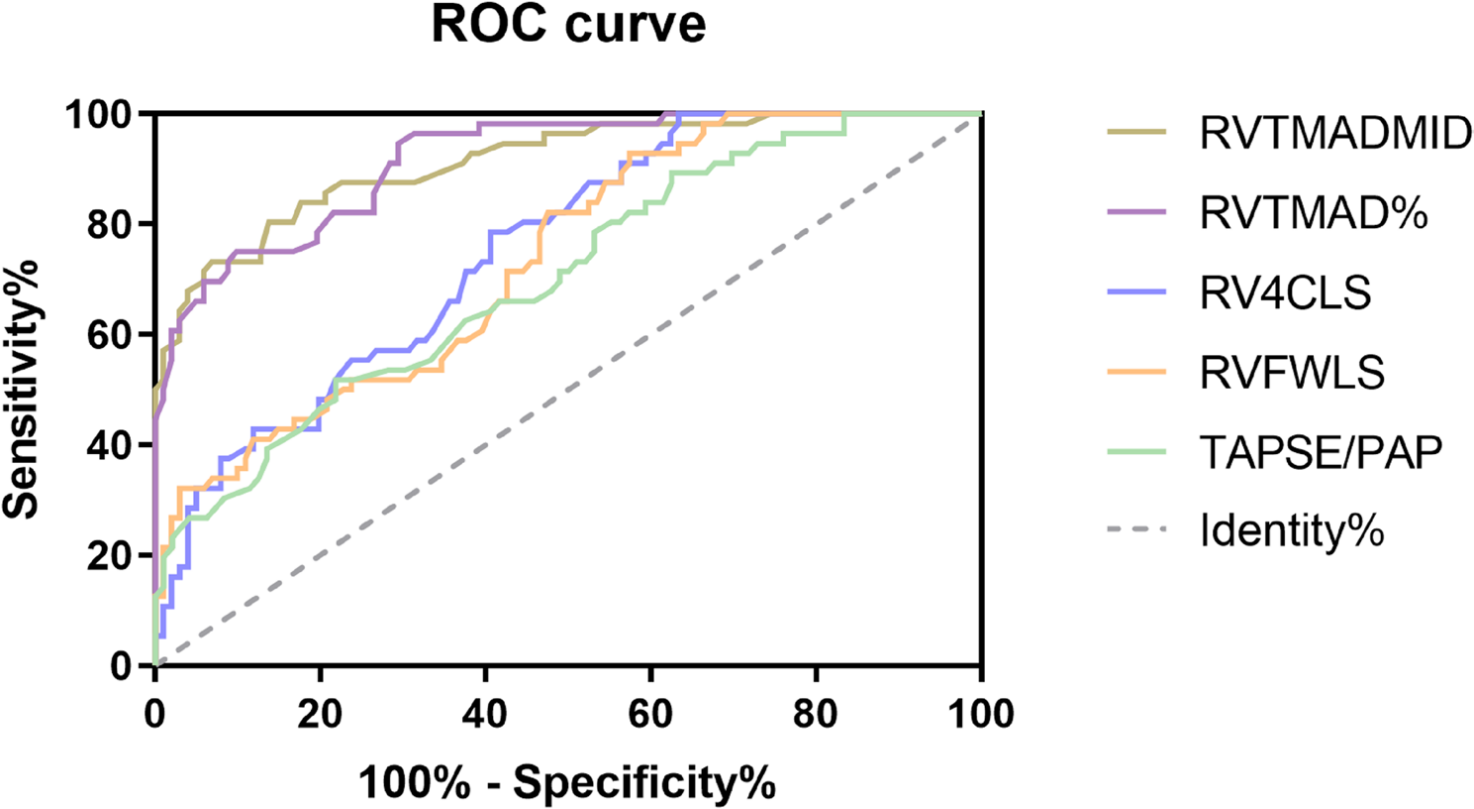
Receiver operating characteristic (ROC) curves evaluating the diagnostic accuracy of RV tissue motion annular displacement (RVTMAD), RV longitudinal strain (RVLS), and tricuspid annular plane systolic excursion (TAPSE)/pulmonary arterial systolic pressure (PASP)for right ventricular dysfunction (RVD) in sepsis

To assess the diagnostic accuracy of STE parameters for detecting RVD, ROC curve analysis was performed (Table 3; Fig. 4). Among all parameters, RVTMADmid and RVTMAD% exhibited the highest diagnostic performances, with AUROC values of 0.913 (95% CI: 0.866–0.960) and 0.917 (95% CI: 0.874–0.960), respectively. The optimal RVTMAD% cutoff of < 16.70% yielded a sensitivity of 94.6% and specificity of 70.6%, while the optimal cutoff of < 10.95 mm for RVTMADmid provided a sensitivity of 80.4% and specificity of 86.3%.

**Table 3:** Area under the receiver operating characteristic (AUROC) curve analysis results for RV tissue motion annular displacement (RVTMAD), RV longitudinal strain (RVLS), and tricuspid annular plane systolic excursion (TAPSE)/pulmonary arterial systolic pressure (PASP)

Variables	AUROC	Cutoff value	95% CI	Sensitivity	Specificity	*P*
RVTMADmid	0.913	10.95	0.866–0.960	80.4	86.3	< 0.001
RVTMAD%	0.917	16.70	0.874–0.960	94.6	70.6	< 0.001
RV4CLS	0.753	−13.25	0.680–0.828	78.6	59.4	< 0.001
RVFWLS	0.735	−18.85	0.656–0.813	92.86	42.6	< 0.001
TAPSE/PASP	0.703	0.52	0.624–0.793	51.8	78.1	< 0.001

*RV4CLS* RV 4-chamber longitudinal strain, *RVFWLS* RV free-wall longitudinal strain, *CI* confidence interval

###  Prognostic value of RVLS and RVTMAD

**Fig. 5:**
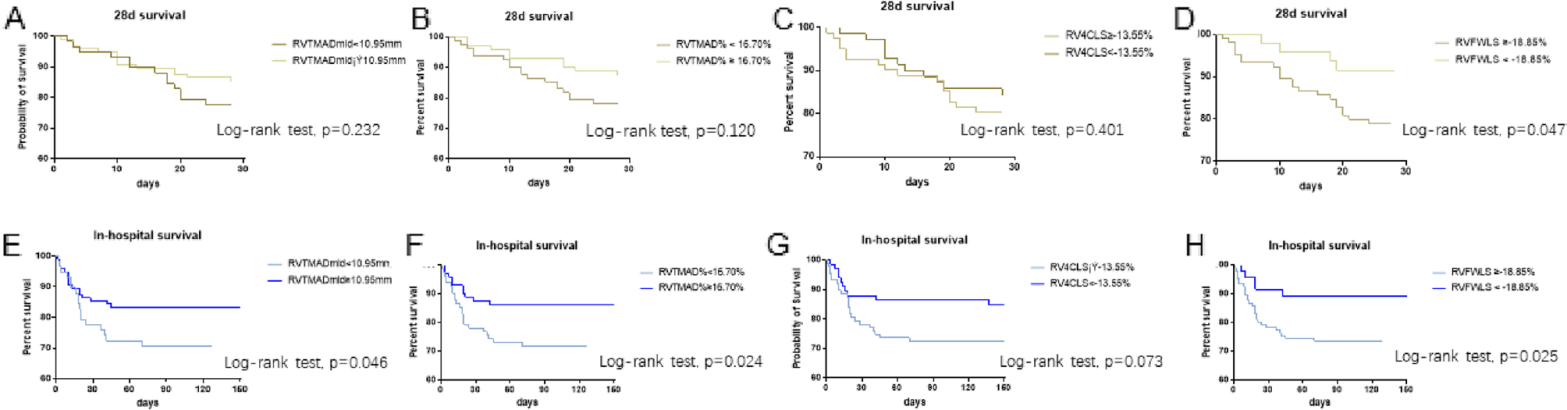
Kaplan-Meier survival curves based on cutoff values of speckle tracking echocardiography (STE) parameters. **A** No significant difference in 28-day mortality was found between patients with RV tissue motion annular displacement (RVTMAD)mid < 10.95 mm and RV tissue motion annular displacement (RVTMAD)mid ≥ 10.95 mm. **B** No significant difference in 28-day mortality was found between patients with RV tissue motion annular displacement (RVTMAD)% < 16.7% and RV tissue motion annular displacement (RVTMAD)% ≥ 16.70%. **C** No significant difference in 28-day mortality was found between patients with RV 4-chamber longitudinal strain (RV4CLS) < −13.55% and RV 4-chamber longitudinal strain (RV4CLS) ≥ −13.55%. **D** Patients with RV free-wall longitudinal strain (RVFWLS) ≥ −18.85% had significantly higher 28-day mortality. **E** Patients with RV tissue motion annular displacement (RVTMAD)mid < 10.95 mm presented significantly higher in-hospital mortality. **F** Patients with RV tissue motion annular displacement (RVTMAD)% < 16.70% presented significantly higher in-hospital mortality. **G** No significant difference in in-hospital mortality was found between patients with RV 4-chamber longitudinal strain (RV4CLS) < −13.55% and RV 4-chamber longitudinal strain (RV4CLS) ≥ −13.55%. **H** Patients with RV free-wall longitudinal strain (RVFWLS) ≥ −18.85% presented significantly higher in-hospital mortality

Kaplan-Meier survival curves, stratified by RVLS and RVTMAD cutoffs, are presented in Fig. 5. Patients with RVTMADmid < 10.95 mm, RVTMAD% < 16.70%, or RVFWLS ≥−18.85% had significantly higher in-hospital mortality. In addition, patients with RVFWLS ≥ −18.85% also exhibited significantly increased 28-day mortality.

Univariate analyses are presented in Tables S2 and S3. To assess potential collinearity among covariates, a correlation matrix was generated. Variables with correlation coefficients greater than 0.3 were carefully reviewed to determine their retention in order to minimize multicollinearity (Tables S4 and S5). Clinically relevant variables with low intercorrelation were subsequently included in the Cox proportional hazards model. The results of the survival analyses based on these models are summarized in Tables S2 and S3. The Cox regression analysis revealed that only SOFA score (HR = 1.072224, 95% CI: 1.006–1.143, *P* = 0.032) was independently associated with in-hospital mortality. Neither RVTMAD nor RVLS retained independent prognostic significance after adjusting for other clinical variables.

## Discussion

This study provides novel evidence supporting the diagnostic utility of STE, particularly RVTMAD, in identifying RVD among patients with sepsis. Our findings demonstrate that both RVTMADmid and RVTMAD% outperform conventional and other STE-derived parameters, including RVLS, in accurately diagnosing RVD with AUROC values exceeding 0.91. In the Cox proportional hazards regression analysis, the SOFA score emerged as the sole independent predictor of in-hospital mortality.

The identification of RVD in septic patients remains clinically challenging due to the right ventricle’s complex anatomy and the hemodynamic variability inherent to sepsis. Conventional echocardiographic indices such as TAPSE and FAC are commonly used but limited by angle dependency, preload sensitivity, and suboptimal imaging conditions in critically ill patients [7, 8, 26]. In contrast, STE-derived indices like RVLS and RVTMAD offer angle-independent assessments with greater reproducibility [11, 14–16]. Our results demonstrated significant correlations between RVTMAD and both TAPSE and FAC, reinforcing its role as a surrogate marker of RV systolic function.

RVLS, particularly RVFWLS, has been validated as a sensitive marker of RV dysfunction in pulmonary hypertension, myocardial infarction, and heart failure [11, 14, 15, 26–28]. In the current study, RVFWLS ≥ − 18.85% was associated with increased short-term mortality, consistent with previous reports [30, 31]. However, RVLS assessment is technically demanding, requiring optimal image quality and clear visualization of the endocardial border, which can be difficult in unstable patients [32]. By contrast, RVTMAD is easier to measure, relying only on the identification of the tricuspid annulus and apex, and is less susceptible to poor image quality [17, 33]. The superior diagnostic performance of RVTMAD over RVLS in our study may be partly explained by the heterogeneous contractile pattern of the RV and the influence of LV function on septal strain measurements. Since RV4CLS includes septal components, it may be confounded in patients with concurrent LV dysfunction [30]. RVTMAD, on the other hand, reflects annular motion and may better isolate RV-specific contractile performance.

RVD in sepsis has been associated with poor clinical outcomes and higher mortality [5, 6]. Our study supports this association, showing that patients with RVD had elevated liver enzymes, higher rates of acute kidney injury, and increased central venous pressure, suggesting systemic venous congestion as a potential pathophysiological mechanism [33–35]. Prior studies have shown that increased right atrial pressure and venous congestion can impair renal and hepatic perfusion, contributing to organ dysfunction [36]. This study demonstrates that STE enables early and sensitive detection of RVD in sepsis, which has important clinical implications. Early recognition of RVD allows for individualized hemodynamic strategies, including judicious fluid resuscitation to avoid volume overload, careful adjustment of ventilator settings to reduce pulmonary vascular resistance, and tailored use of inotropes or pulmonary vasodilators in selected cases. In patients with severe RVD and refractory shock, early consideration of advanced circulatory support may be warranted. These approaches, guided by RVD diagnosis, have the potential to optimize tissue perfusion and improve outcomes.

In the multivariate analysis, only SOFA score remained independently associated with in-hospital mortality, consistent with previous studies [36–40]. Although both RVTMAD and RVLS demonstrated prognostic associations with mortality in univariable analysis, they did not emerge as independent predictors in multivariable models. This may be partly explained by the collinearity between RV function indices and more comprehensive clinical scores such as SOFA, which already reflect global organ dysfunction and systemic severity. Furthermore, RVTMAD and RVLS, while sensitive for early detection of subclinical RV dysfunction, may be more useful as diagnostic or risk stratification tools rather than sole prognostic indicators. Their integration with dynamic clinical variables and composite scores may enhance future mortality prediction models in sepsis.

Several limitations of this study must be acknowledged. First, it was a retrospective single-center study with a modest sample size, which may limit generalizability. Second, only patients with adequate image quality were included, possibly introducing selection bias. Patients with inadequate image quality were excluded from our analysis, potentially resulting in selection bias favoring those with better echocardiographic windows. In further clinical practice and study design, for patients with suboptimal transthoracic echocardiographic images, other diagnostic modalities such as transesophageal echocardiography should be considered. Third, due to the retrospective nature of the study, RV measurements were performed using standard apical four-chamber views rather than RV-focused views, which may underestimate RV dimensions and function. Fourth, potential RV function related confounding factors, such as respiratory mechanics, tricuspid regurgitation severity, and intravascular volume status, were not accounted for. In future prospective studies, we plan to collect these data comprehensively to provide a more precise analysis. Finally, defining the diagnostic time window as within 24 h of ICU admission may have led to classification bias by excluding patients who developed RVD at a later stage of their ICU stay. Using a single early time-point may underestimate the prevalence and impact of late-onset RVD. In future prospective studies, we plan to follow patients from the time of admission throughout the entire treatment process, performing multiple echocardiography follow-ups to obtain more accurate and reliable data. Given these limitations mentioned above, further well-designed prospective, multicenter studies with larger sample sizes are warranted to validate these findings.

## Conclusion

In conclusion, RVTMAD, as a STE-derived parameter, may serve as a reliable tool for the early detection of RVD in septic patients, particularly in cases where conventional parameters are inconclusive or image quality is suboptimal. Given its strong diagnostic performance, RVTMAD may serve as a valuable addition to routine echocardiographic assessments in the ICU setting. Early identification of RVD may guide individualized treatment strategies, including optimization of fluid management, titration of vasoactive agents, and dynamic assessment of RV function, potentially improving hemodynamic stability and clinical outcomes in this vulnerable patient population.

## Supplementary Information

Supplementary Material 1

## Data Availability

The datasets generated and/or analysed during the current study are not publicly available due to containing information that could compromise the privacy of research participants, but are available from the corresponding author on reasonable request.

## References

[1] Evans L, Rhodes A, Alhazzani W, Antonelli M, Coopersmith CM, French C, et al. Surviving sepsis campaign: international guidelines for management of sepsis and septic shock 2021. Crit Care Med. 2021;49(11):e1063–143. 34605781 10.1097/CCM.0000000000005337

[2] Vallabhajosyula S, Jentzer JC, Geske JB, Kumar M, Sakhuja A, Singhal A, et al. New-onset heart failure and mortality in hospital survivors of sepsis-related left ventricular dysfunction. Shock. 2018;49(2):144–9. 28727607 10.1097/SHK.0000000000000952 PMC5766383

[3] Pruszczyk A, Zawadka M, Andruszkiewicz P, LaVia L, Herpain A, Sato R, et al. Mortality in patients with septic cardiomyopathy identified by longitudinal strain by speckle tracking echocardiography: an updated systematic review and meta-analysis with trial sequential analysis. Anaesth Crit Care Pain Med. 2024;43(2):101339. 38128732 10.1016/j.accpm.2023.101339

[4] Winkelhorst JC, Bootsma IT, Koetsier PM, de Lange F, Boerma EC. Right ventricular function and long-term outcome in sepsis: a retrospective cohort study. Shock (Augusta Ga). 2020;53(5):537–43. 31318835 10.1097/SHK.0000000000001413

[5] Vallabhajosyula S, Shankar A, Vojjini R, Cheungpasitporn W, Sundaragiri PR, DuBrock HM, et al. Impact of right ventricular dysfunction on Short-term and Long-term mortality in sepsis: A Meta-analysis of 1,373 patients. Chest. 2021;159(6):2254–63. 33359215 10.1016/j.chest.2020.12.016 PMC8579312

[6] Vallabhajosyula S, Kumar M, Pandompatam G, Sakhuja A, Kashyap R, Kashani K, et al. Prognostic impact of isolated right ventricular dysfunction in sepsis and septic shock: an 8-year historical cohort study. Ann Intensive Care. 2017;7(1):94. 28884343 10.1186/s13613-017-0319-9 PMC5589718

[7] Rudski LG, Lai WW, Afilalo J, Hua L, Handschumacher MD, Chandrasekaran K, et al. Guidelines for the echocardiographic assessment of the right heart in adults: a report from the American society of echocardiography endorsed by the European association of echocardiography, a registered branch of the European society of cardiology, and the Canadian society of echocardiography. J Am Soc Echocardiography: Official Publication Am Soc Echocardiography. 2010;23(7):685–713. quiz 86 – 8. 10.1016/j.echo.2010.05.010 20620859

[8] Hwang JW. Assessment of right ventricular systolic function: conventional methods and modified tricuspid annular plane systolic excursion. J Cardiovasc Imaging. 2019;27(1):34–6. 30701714 10.4250/jcvi.2019.27.e13 PMC6358428

[9] Cersosimo A, Gavazzoni M, Inciardi RM, Radulescu CI, Adamo M, Arabia G, et al. Right ventricle assessment before tricuspid valve interventions. J Cardiovasc Med (Hagerstown). 2024;25(2):95–103. 38149699 10.2459/JCM.0000000000001574 PMC10906196

[10] Haddad F, Hunt SA, Rosenthal DN, Murphy DJ. Right ventricular function in cardiovascular disease, part I: anatomy, physiology, aging, and functional assessment of the right ventricle. Circulation. 2008;117(11):1436–48. 18347220 10.1161/CIRCULATIONAHA.107.653576

[11] Lang RM, Badano LP, Mor-Avi V, Afilalo J, Armstrong A, Ernande L, et al. Recommendations for cardiac chamber quantification by echocardiography in adults: an update from the American society of echocardiography and the European association of cardiovascular imaging. Eur Heart J Cardiovasc Imaging. 2015;16(3):233–70. 25712077 10.1093/ehjci/jev014

[12] van Kessel M, Seaton D, Chan J, Yamada A, Kermeen F, Hamilton-Craig C, et al. Prognostic value of right ventricular free wall strain in pulmonary hypertension patients with pseudo-normalized tricuspid annular plane systolic excursion values. Int J Cardiovasc Imaging. 2016;32(6):905–12. 26931558 10.1007/s10554-016-0862-8

[13] Anwer S, Stollenwerk L, Winkler NE, Guastafierro F, Hebeisen M, Akdis D, et al. Right heart strain in arrhythmogenic right ventricular cardiomyopathy: implications for cardiovascular outcome. Eur Heart J Cardiovasc Imaging. 2024;25(8):1061–8. 38683812 10.1093/ehjci/jeae117 PMC11288757

[14] Serafin A, Kosmala W, Marwick TH. Evolving applications of echocardiography in the evaluation of left atrial and right ventricular strain. Curr Cardiol Rep. 2024;26(6):593–600. 38647564 10.1007/s11886-024-02058-x PMC11199230

[15] Longobardo L, Suma V, Jain R, Carerj S, Zito C, Zwicke DL, et al. Role of Two-Dimensional Speckle-Tracking echocardiography strain in the assessment of right ventricular systolic function and comparison with conventional parameters. J Am Soc Echocardiography: Official Publication Am Soc Echocardiography. 2017;30(10):937–. – 46.e6. 10.1016/j.echo.2017.06.016 28803684

[16] Li Y, Sun C, Zhang L, Zhang Y, Wang J, Zhang J, et al. Feasibility, reproducibility, and prognostic value of fully automated measurement of right ventricular longitudinal strain. J Am Soc Echocardiogr. 2022;35(6):609–19. 35134519 10.1016/j.echo.2022.01.016

[17] Shen T, Picard MH, Hua L, Burns SM, Andrawes MN. Assessment of tricuspid annular motion by speckle tracking in anesthetized patients using transesophageal echocardiography. Anesth Analg. 2018;126(1):62–7. 29116970 10.1213/ANE.0000000000002614

[18] Song J, Yao Y, Lin S, He Y, Zhu D, Zhong M. Feasibility and discriminatory value of tissue motion annular displacement in sepsis-induced cardiomyopathy: a single-center retrospective observational study. Crit Care. 2022;26(1):220. 35851427 10.1186/s13054-022-04095-w PMC9295263

[19] von Elm E, Altman DG, Egger M, Pocock SJ, Gøtzsche PC, Vandenbroucke JP, et al. The strengthening the reporting of observational studies in epidemiology (STROBE) statement: guidelines for reporting observational studies. Lancet. 2007;370(9596):1453–7. 18064739 10.1016/S0140-6736(07)61602-X

[20] Singer M, Deutschman CS, Seymour CW, Shankar-Hari M, Annane D, Bauer M, et al. The third international consensus definitions for sepsis and septic shock (Sepsis-3). JAMA. 2016;315(8):801–10. 26903338 10.1001/jama.2016.0287 PMC4968574

[21] Matthay MA, Arabi Y, Arroliga AC, Bernard G, Bersten AD, Brochard LJ, et al. A new global definition of acute respiratory distress syndrome. Am J Respir Crit Care Med. 2024;209(1):37–47. 37487152 10.1164/rccm.202303-0558WS PMC10870872

[22] Khwaja A. KDIGO clinical practice guidelines for acute kidney injury. Nephron Clinical practice. 2012;120(4):c179-84. 22890468 10.1159/000339789

[23] Bowcock EM, Gerhardy B, Huang S, Orde S. Right ventricular outflow tract doppler flow analysis and pulmonary arterial coupling by transthoracic echocardiography in sepsis: a retrospective exploratory study. Crit Care. 2022;26(1):303. 36192793 10.1186/s13054-022-04160-4 PMC9527734

[24] Lee JH, Park JH. Strain analysis of the right ventricle using two-dimensional echocardiography. J Cardiovasc Imaging. 2018;26(3):111–24. 30310878 10.4250/jcvi.2018.26.e11 PMC6160817

[25] Li Y, Wang Y, Yang Y, Liu M, Meng X, Shi Y, et al. Tricuspid annular displacement measured by 2-dimensional speckle tracking echocardiography for predicting right ventricular function in pulmonary hypertension: a new approach to evaluating right ventricle dysfunction. Medicine (Baltimore). 2018;97(30):e11710. 30045334 10.1097/MD.0000000000011710 PMC6078723

[26] Vallabhajosyula S, Pruthi S, Shah S, Wiley BM, Mankad SV, Jentzer JC. Basic and advanced echocardiographic evaluation of myocardial dysfunction in sepsis and septic shock. Anaesth Intensive Care. 2018;46(1):13–24. 29361252 10.1177/0310057X1804600104

[27] Houard L, Benaets MB, de Meester de Ravenstein C, Rousseau MF, Ahn SA, Amzulescu MS, et al. Additional prognostic value of 2D right ventricular Speckle-Tracking strain for prediction of survival in heart failure and reduced ejection fraction: A comparative study with cardiac magnetic resonance. JACC Cardiovasc Imaging. 2019;12(12):2373–85. 30772232 10.1016/j.jcmg.2018.11.028

[28] Gavazzoni M, Badano LP, Vizzardi E, Raddino R, Genovese D, Taramasso M, et al. Prognostic value of right ventricular free wall longitudinal strain in a large cohort of outpatients with left-side heart disease. Eur Heart J Cardiovasc Imaging. 2020;21(9):1013–21. 31596464 10.1093/ehjci/jez246

[29] D’Souza R, Wang Y, Calderon-Anyosa RJC, Montero AE, Banerjee MM, Ekhomu O, et al. Decreased right ventricular longitudinal strain in children with hypoplastic left heart syndrome during staged repair and follow-up: does it have implications in clinically stable patients? Int J Cardiovasc Imaging. 2020;36(9):1667–77. 32363447 10.1007/s10554-020-01870-0

[30] Carluccio E, Biagioli P, Lauciello R, Zuchi C, Mengoni A, Bardelli G, et al. Superior prognostic value of right ventricular free wall compared to global longitudinal strain in patients with heart failure. J Am Soc Echocardiography: Official Publication Am Soc Echocardiography. 2019;32(7):836–e441. 10.1016/j.echo.2019.02.011 30979539

[31] Muraru D, Onciul S, Peluso D, Soriani N, Cucchini U, Aruta P, et al. Sex- and method-specific reference values for right ventricular strain by 2-dimensional speckle-tracking echocardiography. Circ Cardiovasc Imaging. 2016;9(2):e003866. 26860970 10.1161/CIRCIMAGING.115.003866

[32] Orde S, Huang SJ, McLean AS. Speckle tracking echocardiography in the critically ill: enticing research with minimal clinical practicality or the answer to non-invasive cardiac assessment? Anaesth Intensive Care. 2016;44(5):542–51. 27608336 10.1177/0310057X1604400518

[33] Silverio A, Di Maio M, Scudiero F, Russo V, Esposito L, Attena E, et al. Clinical conditions and echocardiographic parameters associated with mortality in COVID-19. Eur J Clin Invest. 2021;51(12):e13638. 34287861 10.1111/eci.13638 PMC8420215

[34] Geri G, Vignon P, Aubry A, Fedou AL, Charron C, Silva S, et al. Cardiovascular clusters in septic shock combining clinical and echocardiographic parameters: a post hoc analysis. Intensive Care Med. 2019;45(5):657–67. 30888443 10.1007/s00134-019-05596-z

[35] Vallabhajosyula S, Geske JB, Kumar M, Kashyap R, Kashani K, Jentzer JC. Doppler-defined pulmonary hypertension in sepsis and septic shock. J Crit Care. 2019;50:201–6. 30553991 10.1016/j.jcrc.2018.12.008

[36] Padang R, Chandrashekar N, Indrabhinduwat M, Scott CG, Luis SA, Chandrasekaran K, et al. Aetiology and outcomes of severe right ventricular dysfunction. Eur Heart J. 2020;41(12):1273–82. 32047900 10.1093/eurheartj/ehaa037

[37] Lie KC, Lau CY, Van Vinh Chau N, West TE, Limmathurotsakul D. Utility of SOFA score, management and outcomes of sepsis in Southeast Asia: a multinational multicenter prospective observational study. J Intensive Care. 2018;6:9. 29468069 10.1186/s40560-018-0279-7 PMC5813360

[38] Jones AE, Trzeciak S, Kline JA. The sequential organ failure assessment score for predicting outcome in patients with severe sepsis and evidence of hypoperfusion at the time of emergency department presentation. Crit Care Med. 2009;37(5):1649–54. 19325482 10.1097/CCM.0b013e31819def97 PMC2703722

[39] Li W, Wang M, Zhu B, Zhu Y, Xi X. Prediction of median survival time in sepsis patients by the SOFA score combined with different predictors. Burns Trauma. 2020;8:tkz006. 32346543 10.1093/burnst/tkz006 PMC7175770

[40] Dong J, White S, Nielsen K, Banchs J, Wang J, Botz GH, et al. Tricuspid annular plane systolic excursion is a predictor of mortality for septic shock. Intern Med J. 2021;51(11):1854–61. 32618101 10.1111/imj.14957

[41] Zhang H, Chen X, Lian H, Zhang Q, Wang X, Zheng M et al. Prognostic value of tricuspid annular plane systolic excursion and right ventricular outflow tract fractional shortening in mechanically ventilated septic patients. J Cardiothorac Vasc Anesth. 2020. S1053-0770(20)31059-4. 10.1053/j.jvca.2020.10.002. 10.1053/j.jvca.2020.10.002 34756352

