# Supplementary material for: Diagnostic and prognostic value of speckle tracking echocardiography for right ventricular dysfunction in sepsis: a retrospective observational study
Source: BMC Cardiovasc Disord. 2025 Oct 6;25:717. doi: 10.1186/s12872-025-05175-9 (PMC12502397; doi:10.1186/s12872-025-05175-9)
Supplement: Supplementary file 1 — Supplementary Material 1 [file 12872_2025_5175_MOESM1_ESM.docx]

**Table S1.** Intra- and inter-observer reliability for fractional area change (FAC), tricuspid annular plane systolic excursion (TAPSE), right ventricular longitudinal strain (RVLS), and right ventricular tissue motion annular displacement (RVTMAD)

| Variables | ICC | 95% CI | P |
| --- | --- | --- | --- |
| Intra-observer variability |  |  |  |
| FAC | 0.869 | 0.700-0.946 | <0.001 |
| TAPSE | 0.923 | 0.816-0.969 | <0.001 |
| RVLS | 0.893 | 0.749-0.956 | <0.001 |
| RVTMAD | 0.971 | 0.927-0.988 | <0.001 |
| Inter-observer variability |  |  |  |
| FAC | 0.855 | 0.671-0.940 | <0.001 |
| TAPSE | 0.870 | 0.701-0.946 | <0.001 |
| RVLS | 0.864 | 0.688-0.944 | <0.001 |
| RVTMAD | 0.959 | 0.900-0.984 | <0.001 |

ICC: intraclass correlation coefficient; CI: confidence interval.

**Table S2.** Predictors of 28-day mortality using Cox proportional hazards model analysis.

| Variables | Survival (n=127) | Non-survival (n=27) | P | HR (95% CI) | P |
| --- | --- | --- | --- | --- | --- |
| Age (years) | 64.9 ± 1.5 | 68.5 ± 3.8 | 0.315 | - | - |
| Sex (male, %) | 79, 62.2 | 21, 77.8 | 0.182 | - | - |
| APACHE II | 15.9 ± 0.5 | 18.9 ± 1.1 | 0.011 | - | - |
| SOFA | 6.2 ± 0.2 | 8.4 ± 0.6 | <0.0001 | 1.039 (0.968-1.114) | 0.288 |
| cTNT (ng/mL) | 0.06 ± 0.01 | 0.12 ± 0.03 | 0.058 | - | - |
| NT-proBNP (pg/mL) | 2974 ± 498 | 8011 ± 2235 | <0.001 | 1.000 (1.000-1.000) | 0.687 |
| ALT (U/L) | 69.5 ± 10.0 | 179.8 ± 73.9 | <0.007 | - | - |
| AST (U/L) | 105.6 ± 17.5 | 426.9 ± 215.4 | <0.003 | 1.000(1.000-1.001) | 0.144 |
| Cr (µmol/L) | 75.2 ± 4.9 | 87.7 ± 9.1 | 0.102 | - | - |
| LVEF (%) | 57.5 ± 0.8 | 54.4 ± 1.8 | 0.102 | - | - |
| FAC (%) | 41.1 ± 0.9 | 38.5 ± 2.0 | 0.229 | - | - |
| TAPSE | 20.1 ± 0.4 | 15.5 ± 0.8 | 0.008 | 0.972 (0.935-1.010) | 0.151 |
| RVTMADmid (mm) | 12.7 ± 0.4 | 11.1 ± 1.0 | 0.098 | - | - |
| RVTMAD% | 17.1 ± 0.5 | 16.0 ± 1.3 | 0.377 | - | - |
| RV4CLS (%) | -13.7 ± 0.5 | -11.9 ± 0.8 | 0.079 | - | - |
| RVFWLS (%) | -16.5 ± 0.6 | -14.4 ± 1.0 | 0.119 | - | - |
| TAPSE/PASP | 0.77 ± 0.03 | 0.60 ± 0.08 | 0.055 | - | - |

RVLS: right ventricular longitudinal strain; FAC: fractional area change; LVEF: left ventricle ejection fraction; TMAD: tissue motion annular displacement; RVTMAD: right ventricular tricuspid annular motion amplitude displacement; RV4CLS: RV 4-chamber longitudinal strain; RVFWLS: RV free-wall longitudinal strain; APACHE II: acute physiology and chronic health evaluation II; SOFA: sequential organ failure assessment; cTnT: cardiac troponin T; NT-proBNP: N-terminal pro-B type natriuretic peptide; ALT: alanine transaminase; AST: aspartate transaminase; Cr: creatinine; TAPSE: tricuspid annular plane systolic excursion; PASP: pulmonary arterial systolic pressure; HR: hazard ratio.

**Table S3.** Predictors of in-hospital mortality using Cox proportional hazards model analysis.

| Variables | Survival (n=120) | Non-survival (n=34) | P | HR (95% CI) | P |
| --- | --- | --- | --- | --- | --- |
| Age (years) | 64.6 ± 1.5 | 68.9 ± 3.3 | 0.191 | - | - |
| Sex (male, %) | 74, 61.7 | 26, 76.5 | 0.110 | - | - |
| APACHE II | 15.5 ± 0.5 | 19.8 ± 1.0 | <0.001 | - | - |
| SOFA | 6.2 ± 0.2 | 8.1 ± 0.6 | <0.001 | 1.072 (1.006-1.143) | 0.032 |
| cTNT(ng/mL) | 0.06 ± 0.01 | 0.18 ± 0.07 | 0.011 | 1.217 (0.389-3.807) | 0.736 |
| NT-proBNP (pg/mL) | 2690 ± 438 | 7578 ± 1894 | <0.001 | - | - |
| ALT (U/L) | 71.9 ± 10.5 | 149.8 ± 60.4 | 0.039 | - | - |
| AST (U/L) | 107.9 ± 18.4 | 356.6 ± 175.1 | 0.012 | - | - |
| Cr (µmol/L) | 74.4 ± 6.2 | 82.8 ± 9.3 | 0.183 | - | - |
| LVEF (%) | 58.0 ± 0.8 | 53.5 ± 1.8 | 0.011 | 0.995 (0.975-1.015) | 0.631 |
| FAC (%) | 41.5 ± 0.9 | 37.7 ± 1.8 | 0.052 | - | - |
| TAPSE | 20.3 ± 0.4 | 15.7 ± 0.7 | 0.009 | - |  |
| RVTMADmid (mm) | 12.8 ± 0.4 | 11.0 ± 0.9 | 0.035 | 0.986 (0.944-1.030) | 0.522 |
| RVTMAD% | 17.2 ± 0.5 | 15.8 ± 1.3 | 0.228 | - | - |
| RV4CLS (%) | -13.9 ± 0.5 | -11.6 ± 0.7 | 0.016 | - | - |
| RVFWLS (%) | -16.9 ± 0.6 | -13.6 ± 0.9 | 0.007 | 1.004 (0.976-1.032) | 0.802 |
| TAPSE/PASP | 0.77 ± 0.04 | 0.62 ± 0.07 | 0.053 | - | - |

RVLS: right ventricular longitudinal strain; FAC: fractional area change; LVEF: left ventricle ejection fraction; TMAD: tissue motion annular displacement; RVTMAD: right ventricular tricuspid annular motion amplitude displacement; RV4CLS: RV 4-chamber longitudinal strain; RVFWLS: RV free-wall longitudinal strain; APACHE II: acute physiology and chronic health evaluation II; SOFA: sequential organ failure assessment; cTnT: cardiac troponin T; NT-proBNP: N-terminal pro-B type natriuretic peptide; ALT: alanine transaminase; AST: aspartate transaminase; Cr: creatinine; TAPSE: tricuspid annular plane systolic excursion; PASP: pulmonary arterial systolic pressure; HR: hazard ratio.

Table S4. Pairwise Pearson correlation coefficients among candidate variables included in the Cox regression model for 28-day mortality.

| Variables | APACHE II | SOFA | NT-proBNP | ALT | AST | TAPSE |
| --- | --- | --- | --- | --- | --- | --- |
| APACHE II | 1.00 | 0.44 | 0.32 | 0.11 | 0.15 | -0.26 |
| SOFA |  | 1.00 | 0.30 | 0.33 | 0.29 | -0.29 |
| NT-proBNP |  |  | 1.00 | 0.22 | 0.27 | -0.30 |
| ALT |  |  |  | 1.00 | 0.95 | -0.24 |
| AST |  |  |  |  | 1.00 | -0.27 |
| TAPSE |  |  |  |  |  | 1.00 |

APACHE II: acute physiology and chronic health evaluation II; SOFA: sequential organ failure assessment; NT-proBNP: N-terminal pro-B type natriuretic peptide; ALT: alanine transaminase; AST: aspartate transaminase; TAPSE: tricuspid annular plane systolic excursion.

Table S5. Pairwise Pearson correlation coefficients among candidate variables included in the Cox regression model for in-hospital mortality.

| Variables | APACHE II | SOFA | cTNT | NT-proBNP | ALT | AST | LVEF | TAPSE | RVTMADmid | RV4CLS | RVFWLS |
| --- | --- | --- | --- | --- | --- | --- | --- | --- | --- | --- | --- |
| APACHE II | 1.00 | 0.44 | 0.34 | 0.31 | 0.26 | 0.27 | -0.19 | -0.20 | -0.27 | 0.24 | 0.19 |
| SOFA |  | 1.00 | 0.30 | 0.31 | 0.23 | 0.31 | -0.16 | -0.23 | -0.04 | 0.07 | 0.01 |
| cTNT |  |  | 1.00 | 0.54 | 0.34 | 0.24 | -0.30 | -0.29 | -0.23 | 0.19 | 0.19 |
| NT-proBNP |  |  |  | 1.00 | 0.20 | 0.28 | -0.38 | -0.38 | -0.29 | 0.22 | 0.22 |
| ALT |  |  |  |  | 1.00 | 0.78 | -0.11 | -0.09 | -0.20 | -0.05 | -0.09 |
| AST |  |  |  |  |  | 1.00 | -0.20 | -0.20 | -0.24 | -0.06 | -0.09 |
| LVEF |  |  |  |  |  |  | 1.00 | 0.35 | 0.30 | -0.29 | -0.25 |
| TAPSE |  |  |  |  |  |  |  | 1.00 | 0.52 | -0.24 | -0.22 |
| RVTMADmid |  |  |  |  |  | - |  |  | 1.00 | -0.32 | -0.30 |
| RV4CLS |  |  |  |  |  | - |  |  |  | 1.00 | 0.92 |
| RVFWLS |  |  |  |  |  | - |  |  |  |  | 1.00 |

LVEF: left ventricle ejection fraction; RVTMAD: right ventricular tricuspid annular motion amplitude displacement; RV4CLS: RV 4-chamber longitudinal strain; RVFWLS: RV free-wall longitudinal strain; APACHE II: acute physiology and chronic health evaluation II; SOFA: sequential organ failure assessment; cTnT: cardiac troponin T; NT-proBNP: N-terminal pro-B type natriuretic peptide; ALT: alanine transaminase; AST: aspartate transaminase; TAPSE: tricuspid annular plane systolic excursion.
